# LONG-TERM EFFECTS OF MINDFULNESS ON URGE URINARY INCONTINENCE IN OLDER ADULT WOMEN: ANALYZING RCT RESULTS AT 6 MONTHS

**DOI:** 10.1093/geroni/igz038.2786

**Published:** 2019-11-08

**Authors:** Katarina Friberg Felsted

**Affiliations:** University of Utah, Salt Lake City, Utah, United States

## Abstract

Urge urinary incontinence is a condition estimated to cost $82 billion by 2020. Innovative treatments are needed, particularly in the older adult population. A prior combined feasibility study and randomized controlled trial examined six feasibility determinants and five preliminary efficacy outcomes of treating urge urinary incontinence in older adult women (N=25; average age=74 years) utilizing an 8-week mindfulness-based stress reduction (MBSR) intervention compared with the health enhancement program (HEP), which is an active comparison modality specifically validated to be used alongside MBSR in scientific research. Feasibility and preliminary efficacy results were reported at GSA in 2018. This 2019 presentation relays the preliminary efficacy results at 6-month follow up. Outcomes include symptom severity, symptom bother, perceived stress, perceived self-efficacy, and rate and trajectory of change. Future research is needed in the form of a multi-site trial to provide a larger sample with greater diversity.

